# Anti-Inflammatory Effects of GLP-1-Based Therapies beyond Glucose Control

**DOI:** 10.1155/2016/3094642

**Published:** 2016-03-24

**Authors:** Young-Sun Lee, Hee-Sook Jun

**Affiliations:** ^1^Lee Gil Ya Cancer and Diabetes Institute, Gachon University, 7-45 Songdo-dong, Yeonsu-ku, Incheon 406-840, Republic of Korea; ^2^College of Pharmacy and Gachon Institute of Pharmaceutical Science, Gachon University, 7-45 Songdo-dong, Yeonsu-ku, Incheon 406-840, Republic of Korea; ^3^Gachon Medical Research Institute, Gil Hospital, Incheon 405-760, Republic of Korea

## Abstract

Glucagon-like peptide-1 (GLP-1) is an incretin hormone mainly secreted from intestinal L cells in response to nutrient ingestion. GLP-1 has beneficial effects for glucose homeostasis by stimulating insulin secretion from pancreatic beta-cells, delaying gastric emptying, decreasing plasma glucagon, reducing food intake, and stimulating glucose disposal. Therefore, GLP-1-based therapies such as GLP-1 receptor agonists and inhibitors of dipeptidyl peptidase-4, which is a GLP-1 inactivating enzyme, have been developed for treatment of type 2 diabetes. In addition to glucose-lowering effects, emerging data suggests that GLP-1-based therapies also show anti-inflammatory effects in chronic inflammatory diseases including type 1 and 2 diabetes, atherosclerosis, neurodegenerative disorders, nonalcoholic steatohepatitis, diabetic nephropathy, asthma, and psoriasis. This review outlines the anti-inflammatory actions of GLP-1-based therapies on diseases associated with chronic inflammation* in vivo* and* in vitro*, and their molecular mechanisms of anti-inflammatory action.

## 1. Introduction

Glucagon-like peptide-1 (GLP-1) is produced by posttranslational proteolytic cleavage of the* proglucagon* gene product and mainly secreted from the enteroendocrine L cells in the distal intestine in response to nutrient ingestion. GLP-1 is an incretin hormone, which increases glucose-stimulated insulin secretion [1, 2]. GLP-1 is quickly degraded by dipeptidyl peptidase-4 (DPP-4), and inhibition of this proteolytic enzyme enhances its biological half-life [3]. GLP-1 has many beneficial effects on the control of blood glucose levels including stimulation of insulin secretion and inhibition of glucagon secretion, expansion of the beta-cell mass by stimulating beta-cell proliferation and differentiation and inhibiting beta-cell apoptosis, delay of gastric emptying, and reduction of food intake [4–6]. Therefore, GLP-1 has been extensively studied as a possible treatment of type 2 diabetes, and GLP-1 analogues and DPP-4 inhibitors are now widely in clinical use in these patients [7–11].

Expression of the GLP-1 receptor is widely detected in various cells and organs including the kidney, lung, heart, hypothalamus, endothelial cells, neurons, astrocytes, and microglia as well as pancreatic beta-cells [12–17], suggesting that GLP-1 might have additional roles other than glucose-lowering effects. It was reported that GLP-1 shows anti-inflammatory effects on pancreatic islets and adipose tissue, contributing to lowering glucose levels in diabetes [18–20]. In addition to these tissues, emerging data suggest that GLP-1-based therapies also showed anti-inflammatory effects on the liver, vascular system including aorta and vein endothelial cells, brain, kidney, lung, testis, and skin by reducing the production of inflammatory cytokines and infiltration of immune cells in the tissues [17, 21–25]. Thus, GLP-1 therapy may be beneficial for the treatment of chronic inflammatory diseases including nonalcoholic steatohepatitis, atherosclerosis, neurodegenerative disorders, diabetic nephropathy, asthma, and psoriasis [14, 26–32]. Drugs which are GLP-1 receptor agonists or DPP-4 inhibitors are shown in Table 1. In this review, we will introduce some of the chronic inflammatory diseases and then discuss evidence for beneficial effects of GLP-1-based therapies focusing on its anti-inflammatory actions.

## 2. Diabetes

Type 1 diabetes is caused by autoimmune-mediated destruction of pancreatic beta-cells [33], and type 2 diabetes is caused by both insulin resistance and relative deficiency of insulin [34–36]. Inflammation can be a mediator of insulin resistance and beta-cell damage by high glucose, fatty acids, or adipokines released from adipose tissues [37–39]. Thus, inflammation is an important factor for the pathogenesis of both type 1 and type 2 diabetes, and inhibition of inflammation can be a therapeutic strategy for treatment of diabetes.

The proinflammatory cytokines, such as interleukin-1 beta (IL-1*β*), interferon gamma (IFN-*γ*), and tumor necrosis factor alpha (TNF-*α*), inhibit glucose-stimulated insulin secretion and proliferation of beta-cells [40–42]. Treatment of isolated mouse islets with palmitate induced the expression of proinflammatory cytokines TNF-*α*, IL-1*β*, and IL-6. Liraglutide (100 nM), a long-acting GLP-1 analogue, inhibited the palmitate-induced expression of these inflammatory factors and p65 expression [43]. Treatment of cultured human islets with exendin-4 (50 nM), a GLP-1 receptor agonist, suppressed the expression of inflammatory genes such as NF*κ*B1(p105), NF*κ*B2(p100), RelA (also termed p65), TNF receptor superfamily member 1A, and receptor-interacting serine/threonine kinase 2. As well, exendin-4 (50 nM) and cyclic adenosine monophosphate (cAMP) response element-binding protein overexpression additively protected transplanted human islets in streptozotocin- (STZ-) induced diabetic nude mice [44]. Treatment of nonobese diabetic mice with the DPP-4 inhibitor, NVP-DPP728 (30 mg/kg), significantly increased the levels of plasma transforming growth factor beta-1 (TGF-*β*1), an anti-inflammatory cytokine, and increased CD4+CD25+FoxP3+ regulatory T cells, contributing to the remission of diabetes [45]. Treatment of diet-induced obese mice with sitagliptin (4 g/kg), a DPP-4 inhibitor, significantly reduced the expression of inflammatory genes including monocyte chemotactic protein- (MCP-) 1, IL-6, IL-12(p40), IL-12(p35), and IFN-*γ*-induced protein 10 (IP-10) in pancreatic islets and improved glucose-stimulated insulin secretion in isolated islets [19]. Treatment of STZ-induced diabetic rats with another DPP-4 inhibitor, vildagliptin (10 mg/kg), significantly reduced plasma TNF-*α* concentration and decreased nitric oxide concentration in serum and pancreatic homogenates compared with untreated diabetic rats [46]. Treatment with sitagliptin (20 mg/kg) increased serum GLP-1 levels in STZ-induced diabetic monkeys and showed significantly protective effects on STZ-induced islet injury* in vivo* and* in vitro* via activation of the insulin-like growth factor receptor (IGFR)/AKT/mammalian target of rapamycin (mTOR) signaling pathways [47]. These results suggest that GLP-1-based therapies suppress inflammatory cytokines and increase anti-inflammatory mediators in the pancreas.

C-X-C motif chemokine 10 (CXCL10/IP10), which is induced by IFN-*γ*, has an important role in recruiting activated T cells into the islets in type 1 diabetes. Exendin-4 (100 nM) decreased IFN-*γ*-induced signal transducer and activator of transcription-1 (STAT1), which is important for CXCL10 expression in the pancreatic beta-cell line, MIN6 cells, and human islets. Therefore, suppression of CXCL10 production by exendin-4 could reduce islet inflammation by decreasing cytotoxic T lymphocyte recruitment into the islets in autoimmune type 1 diabetes [48].

Serine proteinase inhibitor-9 plays an important role in the survival of cells against attack by natural killer cells and cytotoxic T lymphocytes, which play a direct role in the destruction of pancreatic beta-cells in type 1 diabetes. The GLP-1 receptor agonist, exenatide (a synthetic form of exendin-4) (10 nM), induces the expression of serine protease inhibitor-9 in human islets [49]. These results suggest that GLP-1-based therapies not only directly regulate the expression of inflammatory mediators, but also regulate the recruitment of immunocytes and protect from immunocyte attack, contributing to the preservation of pancreatic islets.

The abundance of proinflammatory cytokines and chemokines in adipose tissue is a key contributor to insulin resistance in type 2 diabetes, and blocking of inflammatory signaling pathways or immune cell infiltration in adipose tissue improves insulin sensitivity [50–52]. Administration of a recombinant adenovirus producing GLP-1 (4 × 10^9^ PFU/mouse) to* ob/ob* mice reduced the macrophage population and production of TNF-*α*, MCP-1, and IL-6 in adipose tissue via inhibition of nuclear factor-kappa B (NF-*κ*B) activation and phosphorylation of ERK1/2 and c-Jun N-terminal kinases [18]. Sitagliptin (4 g/kg) also showed similar effects and reduced the expression of mRNA for inflammatory cytokine genes and macrophage infiltration in adipose tissue of high fat diet- (HFD-) induced obese mice [19]. In patients with type 2 diabetes, sitagliptin (100 mg/day) therapy significantly reduced the plasma levels of C-reactive protein (CRP), IL-6, IL-18, secreted phospholipase-A2, soluble intracellular adhesion molecule- (ICAM-) 1, and E-selectin compared with placebo. The inflammatory score and the homeostatic model assessment index for insulin resistance were significantly reduced in sitagliptin-treated type 2 diabetes patients [7]. Therefore, suppression of inflammatory mediators in adipose tissue by GLP-1-based therapies might contribute to the improvement of insulin sensitivity.

GLP-1-based therapies for diabetes contribute to reduce inflammation and have additional beneficial effects such as islet preservation and improvement of insulin sensitivity in addition to glucose-lowering effects. However, some rare cases of acute pancreatitis and neoplasms have been reported [53–55]; thus the establishment of safety of GLP-1-based therapy should be validated by sufficient further studies.

## 3. Vascular Disease

Inflammation is known to be a risk factor for vascular diseases such as atherosclerosis. Atherosclerotic cardiovascular disease is caused by proinflammatory stimuli in the vascular endothelial cells and is associated with increased plasma levels of TNF-*α*, IL-6, CRP, and circulating endotoxin (i.e., lipopolysaccharide (LPS)) [56, 57]. Atherosclerosis is a chronic inflammatory condition resulting from the invasion and accumulation of white blood cells (foam cells) in the walls of arteries and therefore is a syndrome affecting arterial blood vessels [58].

GLP-1 (5.0 *μ*M) perfusion attenuates LPS-induced microvascular permeability via the cAMP protein kinase A (PKA) pathway [59]. Liraglutide (100 *μ*M) reduced the mRNA expression of adhesion molecules such as vascular cell adhesion molecule- (VCAM-) 1, ICAM-1, and E-selectin in TNF-*α*- or LPS-stimulated human aortic endothelial cells and human umbilical vein endothelial cells [60–62]. Liraglutide (100 nM) induced phosphorylation of calcium/calmodulin-dependent protein kinase I and 5′ adenosine monophosphate-activated protein kinase (AMPK), and inhibition of calcium/calmodulin-dependent protein kinase kinase *β* (CAMKK*β*) abolished the inhibitory effect of liraglutide on the expression of VCAM-1 and E-selectin. In addition, knockdown of AMPK with short hairpin AMPK RNA abolished the liraglutide activation of AMPK and anti-inflammatory effects. These results demonstrate that the anti-inflammatory effects of liraglutide in human aortic endothelial cells is dependent on activation of CAMKK*β* and AMPK, which are cAMP/Ca^2+^ signaling pathways [60]. In addition, it was reported that liraglutide (100 nM) inhibited TNF-*α*- or hyperglyceamia-mediated induction of plasminogen activator inhibitor type-1 in human vascular endothelial cells [23]. Exendin-4 (50 *μ*g/kg/day) treatment resulted in a reduction of atherosclerosis development and the number of monocytes adhering to the endothelium wall in the aortic root in western-type diet-fed APOE^*∗*^3-Leiden.CETP(E3L.CETP) mice [63].

Sitagliptin (25 *μ*M), NVP-DPP728 (270 *μ*M), or liraglutide (1000 ng/mL) treatment significantly reduced oxidized-low-density lipoprotein-induced or PKC activator-induced protein expression of nucleotide-binding domain-like receptor with a pyrin domain 3 (NLRP3), toll-like receptor 4 (TLR4), and IL-1*β* in a human monocytic cell line, THP-1, by decreasing phosphorylated-protein kinase C (PKC) [64]. Administration of linagliptin (10 mg/kg/day), a DPP-4 inhibitor, to ApoE^−/−^ mice, an animal model of atherosclerosis, decreased inflammatory molecule expression and macrophage infiltration in the atherosclerotic aorta [65]. Another report showed that sitagliptin (576 mg/kg) reduced plaque macrophage infiltration and matrix metallopeptidase-9 (MMP-9) levels in ApoE^−/−^ mice [26] and increased activation of AMPK and AKT signaling pathway but inhibited MAPK and ERK1/2 signaling in aorta of ApoE^−/−^ mice [66]. This suggests that sitagliptin has protective actions against atherosclerosis through AMPK and MAPK-dependent mechanisms. In addition, sitagliptin (30 mg/kg/day) and exenatide (3 *μ*g/kg/12 h) significantly inhibited advanced glycation end products-induced oxidative stress in aortic endothelials in high fat diet (HFD)/STZ diabetic rats by reducing endothelin-1 (ET-1) and inflammatory cytokine via RhoA/Rho-associated protein kinase (ROCK)/NF-*κ*B signaling pathways and AMPK activation [67]. Des-fluoro-sitagliptin (200 mg/kg/day) treatment reduced atherosclerotic lesion formation, infiltration of macrophage and T lymphocytes, and the expression of proinflammatory cytokines within plaques in ApoE^−/−^ mice [68]. As well, treatment with alogliptin (20 mg/kg/day), a selective DPP-4 inhibitor, showed similar anti-inflammatory effects in the injured arteries of low-density lipoprotein receptor-deficient mice [69]. Interestingly, metabolite (9-37) of GLP-1 as well as the c-terminal GLP-1 split product (28-37) also reduced plaque inflammation and stabilized atherosclerotic lesions in ApoE^−/−^ mice [70]. These suggest that GLP-1-based therapies have protective effects in atherosclerosis by decreasing macrophage infiltration in atherosclerotic lesions via inhibition of the expression of adhesion molecules.

The loss of sirtuin 6 (SIRT6), which regulates proinflammatory mediators, in human umbilical vein endothelial cells is associated with upregulation of the expression of proinflammatory genes [71]. Liraglutide (100 nM) treatment increased SIRT6 expression and reduced NF-*κ*B expression compared with only high glucose-treated endothelial cells. In diabetic patients treated with GLP-1-based therapy, the protein level of SIRT6 in asymptomatic plaques was significantly increased and TNF-*α* and MMP-9 levels in lesions were significantly reduced compared with diabetic patients without treatment [8]. This result suggests that GLP-1-based therapy has anti-inflammatory effects by induction of SIRT6 expression in endothelial cells.

Cardiovascular disease is increased in type 2 diabetes, and hyperglyceamia is a critical promoter during the development of cardiovascular diseases. Inflammation is an important pathophysiologic factor in diabetic cardiomyopathy. Exendin-4 protects against cardiac contractile dysfunction in an experimental myocardial infarction model. Exendin-4 (5 *μ*g/kg or 1 and 10 nM) inhibited high mobility group box I protein expression, a proinflammatory mediator, in myocardial ischemia and reperfusion in rats [72] and in high glucose-induced myocardial cell injury [73]. Sitagliptin (30 and 50 mg/kg/day) reduced the expression of TNF-*α* and IL-6 in the diabetic heart and had a myocardial protective effect in STZ/HFD-induced diabetic rats [74]. Therefore, GLP-1-based therapy have anti-inflammatory effects on vascular disease and may explain the vasoprotective properties.

## 4. Neurodegenerative Brain Disorder

Neurodegenerative central nervous system disorders are associated with chronic neuroinflammation [75–77]. Epidemiological and clinical studies have suggested a link between type 2 diabetes and Alzheimer's disease [78]. In patients with Alzheimer's disease, insulin receptors and insulin signaling in the brain are desensitized and impaired as found in type 2 diabetes patients. Therefore, drugs used for treatment of diabetes are expected to have a preventive effect against Alzheimer's disease. GLP-1 is known to be produced in the brain [79] and has many functions including neuroprotection [80–82]. In addition, GLP-1 and GLP-1 analogues enter the brain through blood brain barrier [83–86].

The glia may play a critical role in the central nervous system inflammatory responses including Alzheimer's disease, and GLP-1 receptor was observed in astrocytes and microglia [17, 87]. In astrocytes, GLP-1 (1 *μ*M) prevented the LPS-induced IL-1*β* expression by increase of cAMP [17].

Models of Alzheimer's disease include intracerebroventricular injection of STZ [88], intracerebral injection of LPS [88], and the APPSWE/PS1ΔE9 mouse [84]. Exenatide (20 *μ*g/kg/day) treatment inhibited brain TNF-*α* levels, which were induced by intracerebroventricular injection of STZ [89]. GLP-1 (7-36) amide (50 nM) protected the synaptic impairments induced by intracerebral injection of LPS in the rat hippocampus [90]. Liraglutide (25 nmol/kg/day) treatment significantly reduced the inflammatory response in the cortex as measured by the number of activated microglia and prevented degenerative processes in a 7-month-old APP_SWE_/PS1_ΔE9_ mouse model of Alzheimer's disease [86]. In addition, in the 14-month-old APP_SWE_/PS1_ΔE9_ mouse, inflammation was also markedly reduced and restorative effects were improved by liraglutide treatment [91]. The GLP-1 receptor agonist, lixisenatide, exerted neuroprotective effects via reduction of oxidative stress and the chronic inflammation response in the brain of APP_SWE_/PS1_ΔE9_ mouse [92]. In addition, sitagliptin (10 and 20 mg/kg) also showed similar anti-inflammatory effects in APP_SWE_/PS1_ΔE9_ mouse [93]. This suggests that GLP-1-based therapies could have a preventive and restorative effect on the pathophysiology of Alzheimer's disease progression.

Irradiation of the brain causes a chronic inflammatory response. X-ray irradiation of the brain significantly increased IL-6, IL-1*β*, and IL-12p70 cytokine protein expression. Liraglutide (25 nmol/kg/day) treatment reduced the mRNA expression of proinflammatory cytokine genes, which was induced by X-ray irradiation [24].

Parkinson's disease is a chronic and neurodegenerative brain disorder, and inflammatory activity is one of important features of Parkinson's disease. Microglial activation plays a critical role in the pathogenesis of the 1-methyl-4-phenyl-1,2,3,6-tetrahydropyidine- (MPTP-) induced Parkinson's disease model and human Parkinson's disease [27]. Exendin-4 (10 *μ*g/kg) treatment significantly decreased MPTP-induced microglial activation and suppressed MPTP-induced expression of TNF-*α* and IL-1*β* [94]. The inhibitory effect of exendin-4 on microglial activation may have therapeutic potential for the treatment of Parkinson's disease. These anti-inflammatory effects of GLP-1-based therapies on the brain may protect against neurodegenerative central nervous system disorders.

## 5. Nonalcoholic Steatohepatitis

Nonalcoholic steatohepatitis is associated with an inflammation of the liver by an aberrant accumulation of fat in the liver. GLP-1 receptor agonists reduced alanine aminotransferase and aspartate aminotransferase levels in patients with nonalcoholic fatty liver disease (or type 2 diabetes) and improved lipid metabolism and reduced fat mass [21]. Liraglutide (50, 100, and 200 *μ*g/kg/12 h) treatment protected against nonalcoholic fatty liver disease by inhibition of ER stress-associated apoptosis in HFD-fed rats [28]. Liraglutide or exendin-4 (1 nmol/kg/day) treatment dose-dependently reduced steatosis and lobular inflammation in HFD-fed rats or mice compared with the saline-injected group [28, 95], probably due to an increase of SIRT1 [96]. As a matter of fact, exendin-4 (50 *μ*g/kg/day) treatment increased the expression of SIRT1 and its downstream factor, AMPK, in exendin-4 treated mouse livers and hepatocytes. Exendin-4 treatment reduced hepatic expression of the inflammatory markers TNF-*α*, IL-1*β*, and IL-6 and macrophage markers, cluster of differentiation 68 (CD68), and F4/80 in the liver of mice fed a western-type diet [63].

In nonalcoholic steatohepatitis patients with glucose intolerance, liraglutide (0.9 mg/person/day) therapy for 96 weeks resulted in improvement of histological indicators of inflammation in seven subjects out of ten subjects [97]. CRP is produced by the liver and is a marker of inflammation. In a retrospective analysis of 110 obese patients with type 2 diabetes treated with liraglutide, the mean concentration of CRP declined after treatment with liraglutide for a mean duration of 7.5 months [9]. In addition, exenatide plus metformin resulted in a significant reduction in CRP and TNF-*α* compared with baseline [98]. These reports suggest that GLP-1-based therapies improve fatty liver disease by ameliorating inflammation in rodents and humans.

## 6. Nephropathy

Diabetic nephropathy is associated with a state of low-grade inflammation in the microvasculature of the kidney's glomeruli [99, 100]. The GLP-1 receptor is expressed in glomerular capillaries and vascular walls of the mouse kidney [14, 101] and in the glomerulus and proximal convoluted tubules of the rat and pig [29, 102]. GLP-1 receptor deficiency in the diabetic nephropathy-resistant C57BL/6-Akita mouse contributes to the development of diabetic nephropathy, and liraglutide treatment suppressed the progression of nephropathy of the KK/Ta-Akita mouse, which shows high susceptibility to diabetic nephropathy [14], suggesting that GLP-1 action might play an important role in prevention of diabetic nephropathy.

Various studies have shown that GLP-1-based therapies can reduce macrophage infiltration and inflammatory molecules in models of diabetic nephropathy. Exendin-4 (3 and 10 *μ*g/kg/day) treatment significantly downregulated the gene expression of CD14, ICAM-1, and TGF*β*1 in the renal cortex, prevented glomerular macrophage infiltration in glomeruli, and reduced oxidative stress and inflammation in tubular cells in STZ-induced diabetic animals [101, 103]. Treatment with the DPP-4 inhibitor, saxagliptin (10 mg/kg/day), reduced renal tubulointerstitial inflammation by NF-*κ*Bp65-mediated macrophage infiltration in STZ-induced diabetic enos^−/−^ mice [104]. Administration of the DPP-4 inhibitor, PKF275-055 (3 mg/kg/day), or linagliptin in STZ-induced diabetic rats inhibited macrophage infiltration, inflammatory molecules, and NF-*κ*B activity in the glomeruli [105] and significantly reduced glomerular leukocyte infiltration [106]. Sitagliptin (10 mg/kg/day) treatment decreased the expression of proinflammatory cytokine genes IL-1*β* and TNF-*α* in kidney of diabetic ZDF rat [25].

GLP-1-based therapies are also effective in nondiabetic models of kidney injury. In a nondiabetic glomerular injury model, alogliptin (20 mg/kg/day), anagliptin (300 mg/kg/day), or exendin-4 (10 mg/kg) significantly reduced infiltration of CD68-positive inflammatory macrophages in the kidney [107]. In the mouse cisplatin-induced renal injury model, treatment with alogliptin (10 mg/kg/day) significantly decreased cisplatin-induced renal injury via antiapoptotic effects [108]. In addition, after ischemia-reperfusion injury, the expression of proinflammatory cytokines, NF-*κ*B and ICAM-1, as well as macrophage infiltration in the kidney was significantly decreased by exendin-4 (10 *μ*g/kg) or sitagliptin (600 mg/kg) treatment [109]. Therefore, GLP-1-based therapies might be beneficial for nephropathy by reducing glomerular leukocyte infiltration and proinflammatory mediators.

## 7. Other Diseases

Asthma is a chronic pulmonary inflammatory disease. Liraglutide (2 mg/kg) reduced immune cell infiltration and protein expression of E-selectin, TNF-*α*, IL-4, IL-5, and IL-13 in the lung tissue or bronchoalveolar lavage fluid in an ovalbumin-induced chronic asthma model. Liraglutide treatment decreased NF-*κ*B activation, which was reversed by PKA inhibitor, H-89, suggesting that the cAMP-PKA pathway is involved in inhibition of NF-*κ*B activation, and subsequently the inhibition of inflammation [110]. In addition, in mice with bleomycin-induced pulmonary fibrosis, liraglutide treatment inhibited infiltration of immune cells and decreased the content of TGF-*β*1. Liraglutide treatment markedly attenuated bleomycin-induced VCAM-1 and NF-*κ*B activation [30]. These results suggest that GLP-1-based therapies might have beneficial effects on asthma but need to be validated by clinical studies.

Obesity can reduce the quality and count of men's sperm [111, 112]. The expression of TNF-*α*, MCP-1, and F4/80 mRNA levels is increased in the testis and significantly decreased the sperm motility and activity in diet-induced obesity mice, and exenatide (24 nmol/kg/day) treatment suppressed the expression of TNF-*α*, MCP-1, and F4/80 mRNA levels in testis and improved sperm quality in diet-induced obesity mice [111].

In type 2 diabetes patients, GLP-1 and liraglutide also improve clinical symptoms of psoriasis, a skin inflammatory disease, by downregulation of invariant natural killer T cells [31, 113, 114].

GLP-1 (100 nM) or exendin-4 (10 nM) treatment inhibited TNF-*α*-induced expression of receptor for advanced glycation end products (RAGE), ICAM-1, and VCAM-1 in human retinal pigment epithelial cells [32], suggesting that GLP-1-based therapies might have beneficial effects on diabetic retinopathy.

Treatment with the DPP-4 inhibitors, linagliptin (5 mg/kg/day) and sitagliptin (50 mg/kg/day), and the GLP-1 analogue, liraglutide (200 *μ*g/kg/day), significantly reduced inflammatory markers such as inducible NO synthase, cyclooxygenase, and VCAM-1 via the AMPK pathway in LPS-induced endotoxemic shock in rats as a model of human sepsis [115].

These reports suggest that GLP-1-based therapies have anti-inflammatory effects in the lung, testis, skin, and eye.

## 8. Conclusion

Inflammation is a protective process including immune system, vascular system, and molecular mediators. However out-of-control inflammation and chronic inflammation can cause pathological disease. Inflammation is a risk factor for diabetes, atherosclerosis, cardiovascular disease, neurodegenerative central nervous system disorders, nonalcoholic steatohepatitis, and nephropathy.

**Figure 1:**
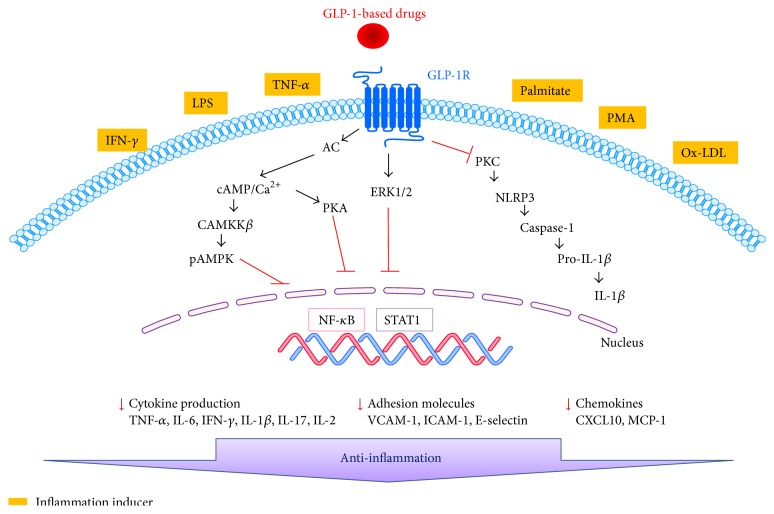
Molecular signals underlying the anti-inflammatory effects of GLP-1-based drugs. DPP-4 inhibitors increase GLP-1 levels in plasma. GLP-1 and GLP-1 receptor (GLP-1R) agonists bind to the GLP-1 receptor, which blocks PKC or NF-*κ*B activation and subsequent expression of NLRP3, IL-1*β*, TNF-*α*, IL-6, VCAM-1, IFN-*γ*, and MCP-1. In addition, GLP-1R signaling activates cAMP/Ca^2+^, CAMKK*β*, and pAMPK, which induces anti-inflammatory effects on monocyte adhesion.

**Figure 2:**
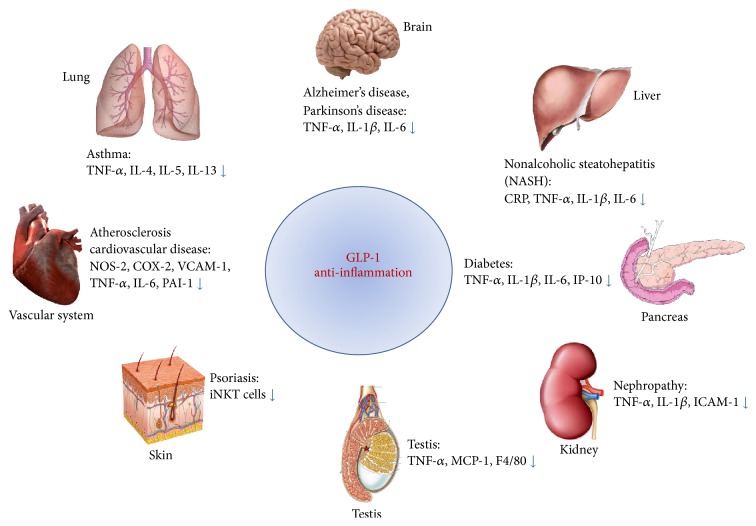
GLP-1-based therapies, including GLP-1, GLP-1R agonists and DPP-4 inhibitors, have anti-inflammatory functions in several organs.

GLP-1-based therapies have many attractive and beneficial effects including their antidiabetic actions on pancreatic beta-cells. However, beyond their metabolic effects, GLP-1-based therapies have been shown to have anti-inflammatory effects via several molecular pathways (Figure 1) in several organs, tissues, and cells (Figure 2). GLP-1-based therapies downregulate proinflammatory responses in inflammatory related diseases. This review concludes that GLP-1-based therapy has beneficial effects on inflammatory disease. Thus GLP-1, GLP-1R agonists, and DPP-4 inhibitors might have important roles as mediators of inflammation.

## Figures and Tables

**Table 1:** GLP-1-based drugs.

GLP-1-based drugs	Generic name	Disease	References
GLP-1 receptor agonists	Exenatide (synthetic form of exendin-4)	Diabetes	[43, 46, 47]
		Vascular disease	[61, 65, 70]
		Nonalcoholic steatohepatitis	[61, 93, 96]
		Nephropathy	[99, 101, 105, 107]
		Neurodegenerative brain disorder	[87, 92]
		
	Liraglutide	Diabetes	[48]
		Vascular disease	[8, 23, 58–60]
		Neurodegenerative brain disorder	[24, 84, 89]
		Nonalcoholic steatohepatitis	[9, 28, 95]
		Nephropathy	[14]
		Asthma	[30, 108]
		Psoriasis	[31, 111, 112]
		
	Lixisenatide	Neurodegenerative brain disorder	[90]
		
	Albiglutide		
	Taspoglutide		
	Dulaglutide		

DPP-4 inhibitors	Sitagliptin	Diabetes	[7, 19, 45]
		Vascular disease	[26, 64, 65, 72]
		Neurodegenerative brain disorder	[91]
		Nephropathy	[25, 107]
		
	Des-fluoro-sitagliptin	Vascular disease	[66]
		
	Alogliptin	Vascular disease	[67]
		Nephropathy	[105, 106]
		
	Linagliptin	Nephropathy	[104]
		Vascular disease	[63]
		
	Vildagliptin (PKF-275-055)	Diabetes	[49]
		Nephropathy	[103]
		
	NVP-DPP728	Diabetes	[44]
		
	Anagliptin	Nephropathy	[105]
		
	Saxagliptin	Nephropathy	[102]

## Abbreviations

AMPK: 5′ adenosine monophosphate-activated protein kinase
CAMKK*β*: Calcium/calmodulin-dependent protein kinase kinase *β*
CD: Cluster of differentiation
CRP: C-reactive protein
CXCL10: C-X-C motif chemokine 10
cAMP: Cyclic adenosine monophosphate
DPP-4: Dipeptidyl peptidase-4
Erk: Extracellular signal-regulated kinase
GLP-1: Glucagon-like peptide-1
HFD: High fat diet
ICAM: Intercellular adhesion molecule
IFN: Interferon
IL: Interleukin
LPS: Lipopolysaccharide
MCP-1: Monocyte chemotactic protein
MPTP: 1-Methyl-4-phenyl-1,2,3,6-tetrahydropyidine
NF-*κ*B: Nuclear factor-kappa B
NLRP3: Nucleotide-binding domain-like receptor with a pyrin domain 3
PKA: Protein kinase A
PKC: Protein kinase C
SIRT: Sirtuin
STZ: Streptozotocin
TLR4: Toll-like receptor 4
TGF-*β*: Transforming growth factor beta
TNF-*α*: Tumor necrosis factor alpha
VCAM: Vascular cell adhesion molecule.
